# Elevated levels of IRF1 and CASP1 as pyroptosis-related biomarkers for intestinal epithelial cells in Crohn’s disease

**DOI:** 10.3389/fimmu.2025.1551547

**Published:** 2025-02-13

**Authors:** Xiaofang Xu, Xiaodan Lv, Ruizhi Zeng, Zhixi Huang, Ziqian Huang, Bing Han, Guangfu Lin, Jianing Lin, Shiquan Li, Junhua Fan, Xiaoping Lv

**Affiliations:** ^1^ Department of Gastroenterology, The First Affiliated Hospital of Guangxi Medical University, Nanning, China; ^2^ Department of Gastroenterology, The Affiliated Tumor Hospital of Guangxi Medical University, Nanning, China

**Keywords:** biomarkers, pyroptosis, intestinal epithelial cells, Crohn’s disease, bulk RNA sequencing, single-cell RNA sequencing

## Abstract

**Introduction:**

Crohn’s disease (CD) is a complex inflammatory condition with the potential for severe complications. Pyroptosis is an inflammatory form of programmed cell death, and the role of pyroptosis in intestinal epithelial cells of CD remains unclear.

**Methods:**

In this study, pyroptosis-related hub genes were identified using datasets from the Gene Expression Omnibus database through differential expression analysis, machine learning algorithms, and single-cell sequencing analysis. Hub gene expression was validated using clinical samples and a trinitrobenzene sulfonic acid (TNBS)-induced colitis rat model.

**Results:**

Six pyroptosis-related hub genes (*CASP1, IRF1, ZBP1, MLKL, MMP1, HTRA1*) were identified. *IRF1* and *CASP1* exhibited significant upregulation in CD, including both colonic and ileal subtypes, with good diagnostic value across different CD subtypes. Additionally, these two genes were not elevated in any other intestinal disorders, except for ulcerative colitis. Single-cell sequencing analysis revealed a significant interaction between intestinal epithelial cells (IECs) and monocytes. The clinical samples further confirmed that the mRNA levels of *IRF1* and *CASP1* were significantly higher in CD patients compared to healthy controls. Additionally, the colitis rat model validated the upregulation of Irf1 and Casp1 at both mRNA and protein levels.

**Conclusion:**

Our findings identified *IRF1* and *CASP1* as critical pyroptosis-related biomarkers for IECs in CD, contributing to the understanding of pyroptosis in CD pathogenesis.

## 1 Introduction

Crohn’s disease (CD) is a form of inflammatory bowel disease (IBD) that can involve all parts of the gastrointestinal tract (1, 2). The global incidence and prevalence of CD are increasing, resulting in a significant socio-economic burden (3). The etiology involves environmental factors, genetic predisposition, mucosal barrier dysfunction, and impaired immune response (4). Despite this understanding, the precise mechanism of CD remains unclear.

Pyroptosis, a form of programmed cell death carried out by the gasdermin (GSDM) protein family, is characterized by NOD-like receptor pyrin domain containing 3 (NLRP3) activation, gasdermin D (GSDMD) protein cleavage, and the formation of cell membrane pores (5). This process plays a critical role in the host’s defense against pathogens and the regulation of inflammatory responses (6). However, excessive pyroptosis results in the release of numerous inflammatory mediators, culminating in cell death, tissue damage, and potentially precipitating autoimmune inflammation or septic shock (7). Increasing evidence demonstrates that excessive pyroptosis is implicated in the pathogenesis of CD. In our previous study, we found that the pyroptosis-related proteins Caspase-1 (CASP1), GSDMD, and NLRP3 were elevated in colonic biopsies of CD patients compared with controls (8). Gong et al. demonstrated that NLRP3/Caspase-1-driven macrophage pyroptosis plays a significant role in active CD, and inhibiting NLRP3 and Caspase-1 with drugs alleviates experimental colitis (9). However, while these findings suggest an important role for pyroptosis in CD, most studies have focused on macrophages and immune cells (10), with limited research exploring the role of pyroptosis in intestinal epithelial cells (IECs), which are pivotal for maintaining intestinal homeostasis and mucosal integrity.

As the first line of defense in the intestine, IECs play a central role in pathogen resistance and immune regulation. Recent studies demonstrated that pyroptosis can also occur in the intestinal epithelium (11). GSDME-mediated pyroptosis in IECs has been described as being correlated with abnormal mucosal inflammation in CD and promotes colitis by releasing proinflammatory cytokines (12). Furthermore, Osterman et al. demonstrated that pyroptosis in ileal epithelial cells could serve as a predictor for the therapeutic response to vedolizumab in CD (13). Despite these early findings, the research on pyroptosis-related biomarkers in IECs in CD is limited, and the specific molecular mechanisms by which pyroptosis in IECs contributes to the initiation of inflammation in CD are not yet fully elucidated.

This study aims to explore the hub genes and pathways associated with pyroptosis in IECs of CD patients. Transcriptomics and single-cell RNA sequencing data were utilized, and machine-learning algorithms were applied to identify hub genes and investigate their biological functions. The expression patterns of these hub genes were further validated using clinical samples and a trinitrobenzene sulfonic acid (TNBS)-induced colitis rat model. Our findings are expected to provide novel insights into potential therapeutic and diagnostic targets for CD.

## 2 Materials and methods

### 2.1 Expression profile data processing and determination of pyroptosis-related genes (PRGs)

Training dataset (GSE75214) and validation datasets (GSE20881, GSE52746, GSE6731, GSE37013, GSE23750, GSE159008, GSE65107, GSE1484) were downloaded from the database of the Gene Expression Omnibus (GEO) (https://www.ncbi.nlm.nih.gov/geo/). The training dataset GSE75214 consisted of 75 individuals with active CD and 22 controls. Details of the GEO accession IDs and sample information for each dataset are summarized in **Supplementary Table S1**. The dataset cohort normalization was conducted using the limma package (14). PRGs were extracted from GeneCards (https://www.genecards.org/) by using “pyroptosis” as the keyword, and 253 protein-coding PRGs were screened out with correlation scores > 1 (**Supplementary Table S2**).

### 2.2 Differentially expressed PRGs (DE-PRGs) identification and functional enrichment analysis

The expression data for 227 PRGs were initially retrieved from the GSE75214 dataset for the control and CD samples. DE-PRGs were screened with the limma package, and *p* < 0.05 and |log2 fold change (FC)| > 0.5 were considered statistically significant. The DE-PRGs expression heatmap was constructed with the pheatmap package, while volcano plots were created using the ggplot2 package. The ClusterProfiler package was used in the Gene Ontology (GO) and Kyoto Encyclopedia of Genes and Genomes (KEGG) (www.kegg.jp/kegg/kegg1.html) pathway enrichment analyses of the DE-PRGs.

### 2.3 CD pyroptosis-related hub genes identification and validation

The pyroptosis-related hub genes in CD were screened with LASSO logistic regression (15) and the random forest (RF) algorithm (16) based on the DE-PRGs. The LASSO logistic regression used the glmnet package, while the RF algorithm was conducted using the randomForest package. The overlapping genes from the two algorithms were selected as hub genes using the Venn package, and the hub gene expression patterns were validated in GSE75214. The Pearson correlation between hub genes in the CD group was calculated and visualized using the corrplot package. Subsequently, the differential expression of hub genes was verified using the validation dataset GSE20881.

### 2.4 Single-cell data processing

The GSE164985 dataset was downloaded from GEO, containing three CD samples and four control colonic epithelial cell samples. The single-cell data was read and filtered with the Seurat package (17) according to the following standards: 200 < single-cell gene count < 6000, unique molecular identifier (UMI) > 1000, and mitochondrial gene expression < 20% (UMI count). Gene expression normalization and scaling were conducted with the LogNormalize function, and variance-stabilizing transformation was used to identify highly variable genes (HVGs). Batch effects were eliminated with the Harmony package (18), and samples were integrated using the merge function (17). The optimal number of principal components (PCs) was determined using the Seurat ElbowPlot function. The t-distributed stochastic neighbor embedding (t-SNE) analysis involved 30 PCs. Cell clusters were identified using the FindClusters function with a clustering resolution of 1.2. Cell types were annotated using the SingleR package (19). Then, the expression of hub genes among the different cell clusters was verified.

### 2.5 Cell communication analysis

Cell-cell communication was analyzed using the CellChat package and CellChatDB.human (V1.6.0) as a reference (20). To examine whether there were significant interactions between the two cell types, the source cell type marker genes were searched against those of the target cell type according to the CellChatDB ligand-receptor pairs.

### 2.6 Diagnostic capabilities of hub genes in CD and its subtypes, and expression in other inflammatory diseases

Expression profiles of different types of CD were analyzed in the training dataset (GSE75214) and the validation datasets (GSE20881, GSE52746) using the “limma” package. The diagnostic value of hub genes for CD and its subtypes was evaluated with the “pROC” packages, and receiver operating curves were plotted to assess model performance. Additionally, hub gene expression was examined in datasets of other intestinal inflammations [ulcerative colitis (UC), infectious colitis, intestinal ischemia-reperfusion injury, amebic colitis, collagenous colitis, lymphocytic colitis, irritable bowel syndrome (IBS)] from the GSE6731, GSE37013, GSE23750, GSE159008, GSE65107, and GSE1484 datasets.

### 2.7 Participant selection and colonic biopsy collection

Colon biopsies were collected during colonoscopy from healthy controls (n = 12) and patients with CD (n = 12) from the First Affiliated Hospital of Guangxi Medical University. The inclusion criteria for CD patients were adults diagnosed with CD based on established diagnostic criteria as previously reported (21). Exclusion criteria for CD patients included those who were critically ill, required immediate surgical intervention, or were pregnant. According to the Crohn’s Disease Activity Index (CDAI) score (21), CD patients were divided into remission (CDAI < 150), mild disease activity (CDAI 150-220), moderate disease activity (CDAI 221-450), and severe disease activity (CDAI > 450). Biopsies were taken from inflamed areas in active CD and from previously inflamed areas or areas still inflamed in remission CD (22). The healthy colon mucosa was obtained from adult individuals with hemorrhoids or polyps, or from those who underwent routine colonoscopy, provided that they had no autoimmune or inflammatory diseases and were not pregnant (23, 24). The sample size of this study was based on works in previous publications (9). The study was approved by the Ethics Committee of The First Affiliated Hospital of Guangxi Medical University (Approval Number: 2023-E685-01) and strictly followed the Declaration of Helsinki. All participants provided their written informed consent to participate in this study. Detailed information about the study procedures, risks, and benefits was provided to all participants, and they were allowed to ask questions and withdraw at any time without penalty. Colon biopsies were used for subsequent real-time quantitative polymerase chain reaction (RT-qPCR) analysis.

### 2.8 TNBS-induced colitis in rats

**Figure 1:**
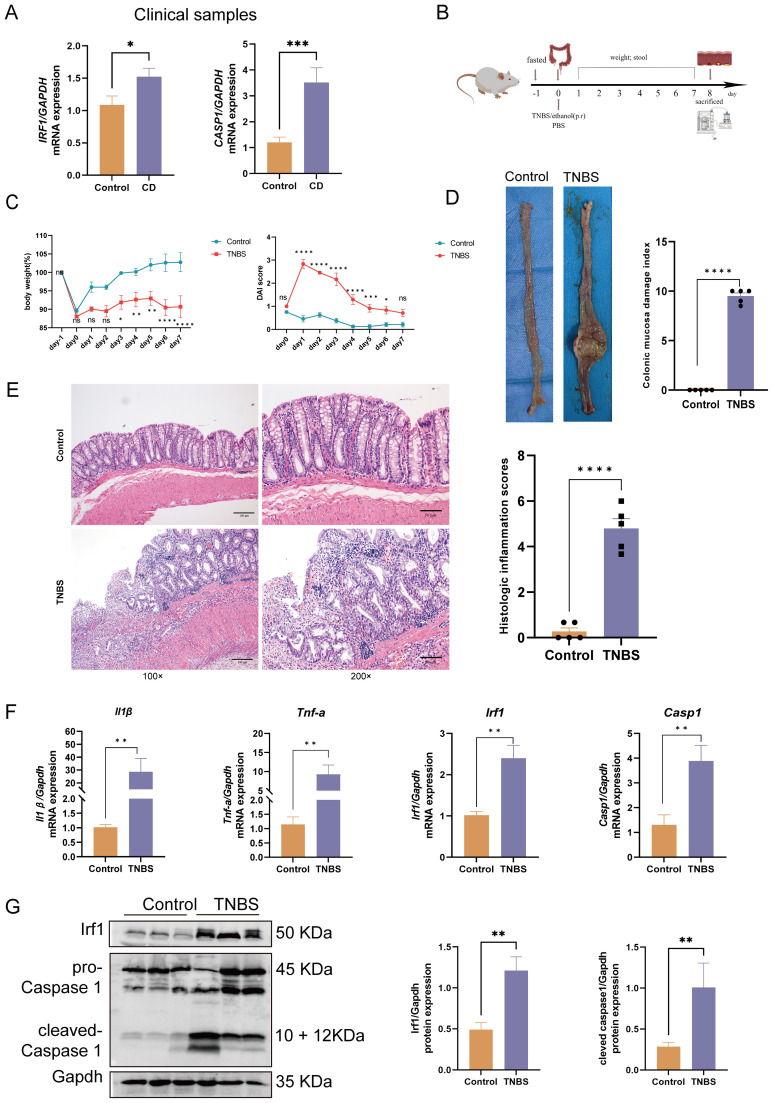
Clinical and animal experimental validation of hub genes. **(A)** *IRF1* and *CASP1* mRNA expression in CD patients versus healthy controls. **(B)** TNBS-induced colitis experimental design. **(C)** Daily rat body weight changes (n = 8); daily changes in rat DAI (n = 8). **(D)** Macroscopic colon appearance and CMDI score in rats (n = 5). **(E)** H&E staining of rat colons (100× and 200× magnification) and histopathological scores (n = 5). Scale bars represent 100 μm and 50 μm. **(F)** The mRNA expression of *Tnf-α, Il-1β, Irf1*, and *Casp1* was significantly upregulated in the colitis rats compared with the controls (n = 6). **(G)** The protein expression of Irf1 and Casp1 was significantly upregulated in the colitis rats compared with the controls (n = 6 or 5). Data represent the means ± SEM. CD, Crohn’s disease; TNBS, trinitrobenzene sulfonic acid; DAI, disease activity index; CMDI, colonic mucosa damage index; H&E, hematoxylin and eosin; SEM, standard error of the mean ^ns^ *p* > 0.05, *p < 0.05, **p < 0.01, ***p < 0.001, ****p < 0.0001.

Male Sprague-Dawley rats (180-210 g, Beijing Viton Lever Experimental Animal Center, Beijing, China) were housed in a specific pathogen-free environment at the Guangxi Medical University Experimental Animal Center. During the experiments, the rats had ad libitum access to food and water and maintained a 12-h light/dark cycle. All animal experiments were approved by the Ethics Committee of Guangxi Medical University (Approval Number: NO.202302001). These experiments were conducted in accordance with the China Laboratory Animal Guidelines for Ethical Review of Animal Welfare (GB/T35892-2018) and Guiding Opinions on the Treatment of Laboratory Animals. The sample size calculation was conducted using the resource equation method (25). The rats were randomly assigned to control and TNBS groups (n = 8 rats per group). The colitis model induced by TNBS replicates the clinical characteristics of human CD (26). Colitis was induced by TNBS based on the method described by Hoffmann et al. (27) with minimal changes. Briefly, rats were fasted for 24 hours and anesthetized with isoflurane. Then, 100 mg/kg TNBS (Sigma Aldrich) dissolved in 0.25 ml 50% ethanol (v/v) was administered to the rats by enema. Control rats received 0.5 ml phosphate-buffered saline. **Figure 1B** depicts the modeling process. After the enema, the rats were maintained in a head-down position for one minute and kept warm until they recovered from anesthesia. They were then returned to their cages.

The rat statuses were examined by general examination and specifically via the disease activity index (DAI), which is a combined score for diarrhea, weight loss, and hematochezia (28). On day 8, the rats were euthanized under anesthesia with a 5% isoflurane overdose followed by cervical dislocation. The colons were excised and photographed. The colon mucosal damage index (CMDI) was assessed in accordance with previously reported criteria (29). Some colon tissue samples were fixed in 4% paraformaldehyde and used for histopathological analysis through Hematoxylin and Eosin (H&E) staining. The remaining tissues were analyzed with RT-qPCR and western blot.

### 2.9 H&E staining

The fixed rat colon tissues were embedded in paraffin and sectioned into 5-μm slices. After deparaffinization and rehydration, the sections were stained with a HE Stain Kit (Solarbio, G1120, China). Images were captured using an Olympus microscope (Olympus, Japan). Histological scoring was performed based on previously published criteria (30).

### 2.10 RT-qPCR

Total RNA was extracted from the colon tissues using RNAiso Plus (Takara, Tokyo, Japan) following the manufacturer’s instructions. Subsequently, the extracted RNA (1 µg) was reverse-transcribed using a complementary DNA synthesis kit (Vazyme, Nanjing, China, cat. no. R323). Real-time qPCR was conducted using an ABI 7500 Real-Time PCR System (Applied Biosystems, Foster City, CA, USA) and ChamQ Universal SYBR qPCR Master Mix (Vazyme, cat. no. Q711). The TNF-α, IL-1β, IRF1, CASP1, and GAPDH primers were synthesized by Sangon Biotech (Shanghai, China). **Supplementary Table S3** lists the sequences and associated target species. The FC was determined by the comparative threshold cycle (2^-ΔΔCt^) relative quantification method.

### 2.11 Western blot

Total protein was extracted from the rat colon tissue using RIPA Lysis Buffer supplemented with protease inhibitor (Solarbio, Beijing, China), according to the manufacturer’s instructions. Proteins were subjected to SDS-PAGE gels and were then transferred to PVDF membranes. After blocking with 5% non-fat milk at room temperature for 1 h, the membranes were incubated with rabbit anti-CASP1 (1:1000, ab179515, Abcam) or anti-IRF1 (1:1000, R24756, ZenBio) primary antibody overnight at 4°C. After washing, membranes were incubated with goat-anti-rabbit DyLight 800 (1:10000, Thermo Fisher Scientific) for 1 h at room temperature before detection using a Li-Cor Odyssey CLx (Li-Cor).

### 2.12 Statistical analysis

Transcriptome and single-cell sequencing data were processed using RStudio software (RStudio, Boston, Massachusetts). In both clinical and animal experiments, comparisons between two groups were made using an unpaired Student’s t-test or Mann-Whitney test, depending on the data distribution. For comparisons involving more than two groups, a two-way ANOVA with Sidak’s *post-hoc* test was applied. Data from clinical and animal experiments are presented as the mean ± standard error of the mean (SEM). All replicates (n) from each experiment were analyzed using Prism 8 (GraphPad Software, La Jolla, CA, USA). Statistical significance was determined at *p* < 0.05.

## 3 Results

### 3.1 Identification of DE-PRGs and enrichment analysis

**Figure 2:**
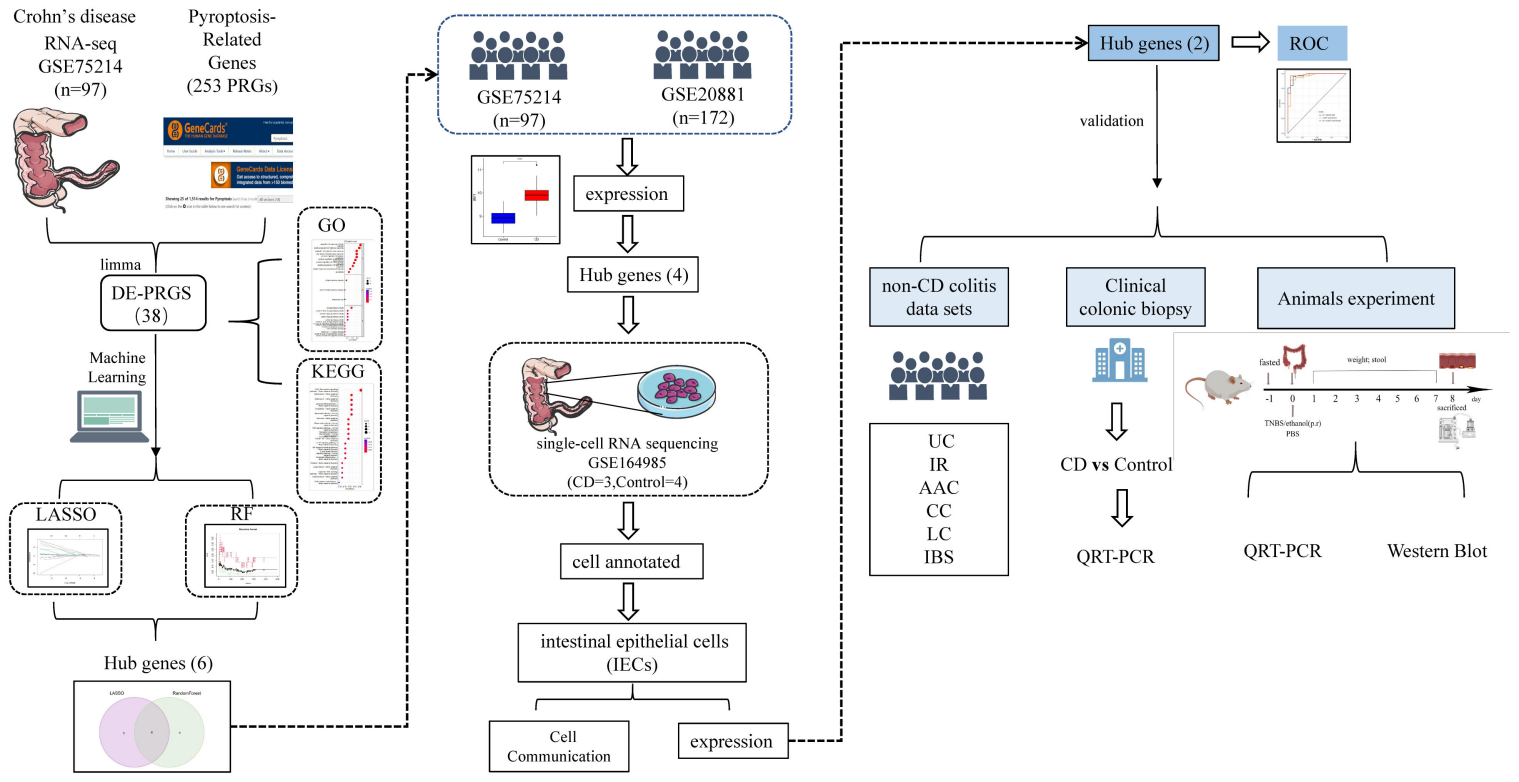
Study design flow chart.

**Figure 3:**
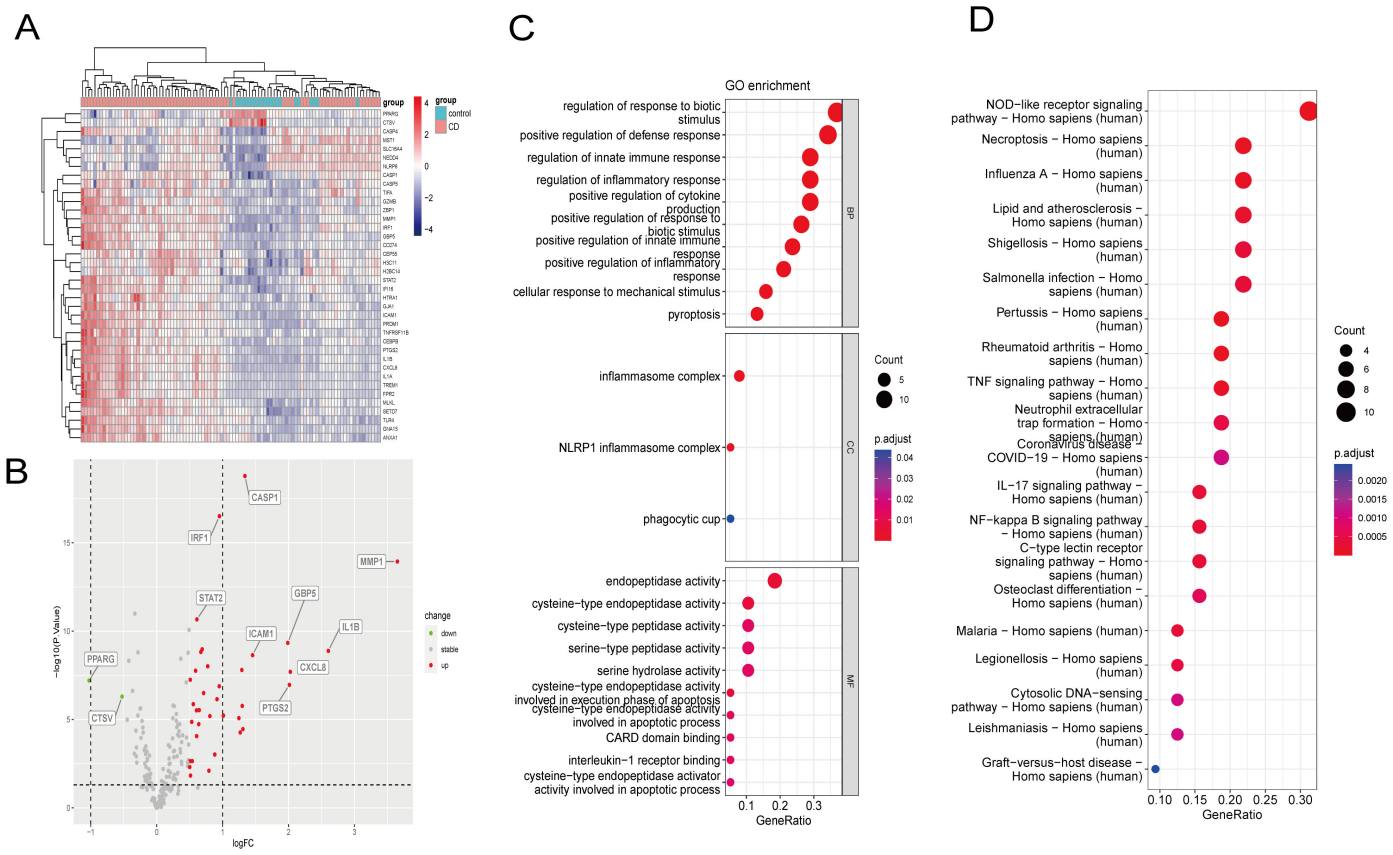
Identification of DE-PRGs and enrichment analysis of the GSE75214 dataset. **(A)** DE-PRGs heatmap. X-axis: sample size; Y-axis: DE-PRGs. **(B)** Volcano plot of DE-PRGs in the GSE75214 dataset between CD patients and healthy controls. X-axis: logFC; Y-axis: –logP.value. **(C)** DE-PRGs GO analysis results. **(D)** DE-PRGs KEGG analysis results. DE-PRGs, differentially expressed pyroptosis-related genes; GO, Gene Ontology; KEGG, Kyoto Encyclopedia of Genes and Genomes.

The flow chart in **Figure 2** depicts the study design. The cluster plot and principle component analysis (PCA) score plots indicated good intra-group similarity and distinct distribution between the CD group and controls (**Supplementary Figures S1A, B**). In total, 38 out of 227 PRGs (2 downregulated and 36 upregulated) were differentially expressed between CD and controls in the GSE75214 dataset (**Supplementary Table S4**). The DE-PRGs expression patterns were visualized in a clustering heatmap (**Figure 3A**) and volcano map (**Figure 3B**), with the majority of DE-PRGs showing upregulation in CD.

GO and KEGG functional enrichment analyses identified the top 10 enriched terms (**Figures 3C, D**). GO analysis demonstrated that the terms associated with the cellular component were inflammasome complex, NLRP1 inflammasome complex, and phagocytic cup. GO molecular function analysis determined that the main enriched terms were endopeptidase activity, cysteine-type peptidase activity, cysteine-type endopeptidase activity, serine-type peptidase activity, and serine hydrolase activity. In the biological process, the most enriched annotations were responses to biotic stimulus regulation, positive defense response regulation, innate immune response regulation, inflammatory response regulation, and positive cytokine production regulation. The enriched KEGG pathways were those of NOD-like receptor signaling, necroptosis, influenza A, lipid and atherosclerosis, and shigellosis.

### 3.2 Identification and validation of six hub genes in the transcriptome dataset

**Figure 4:**
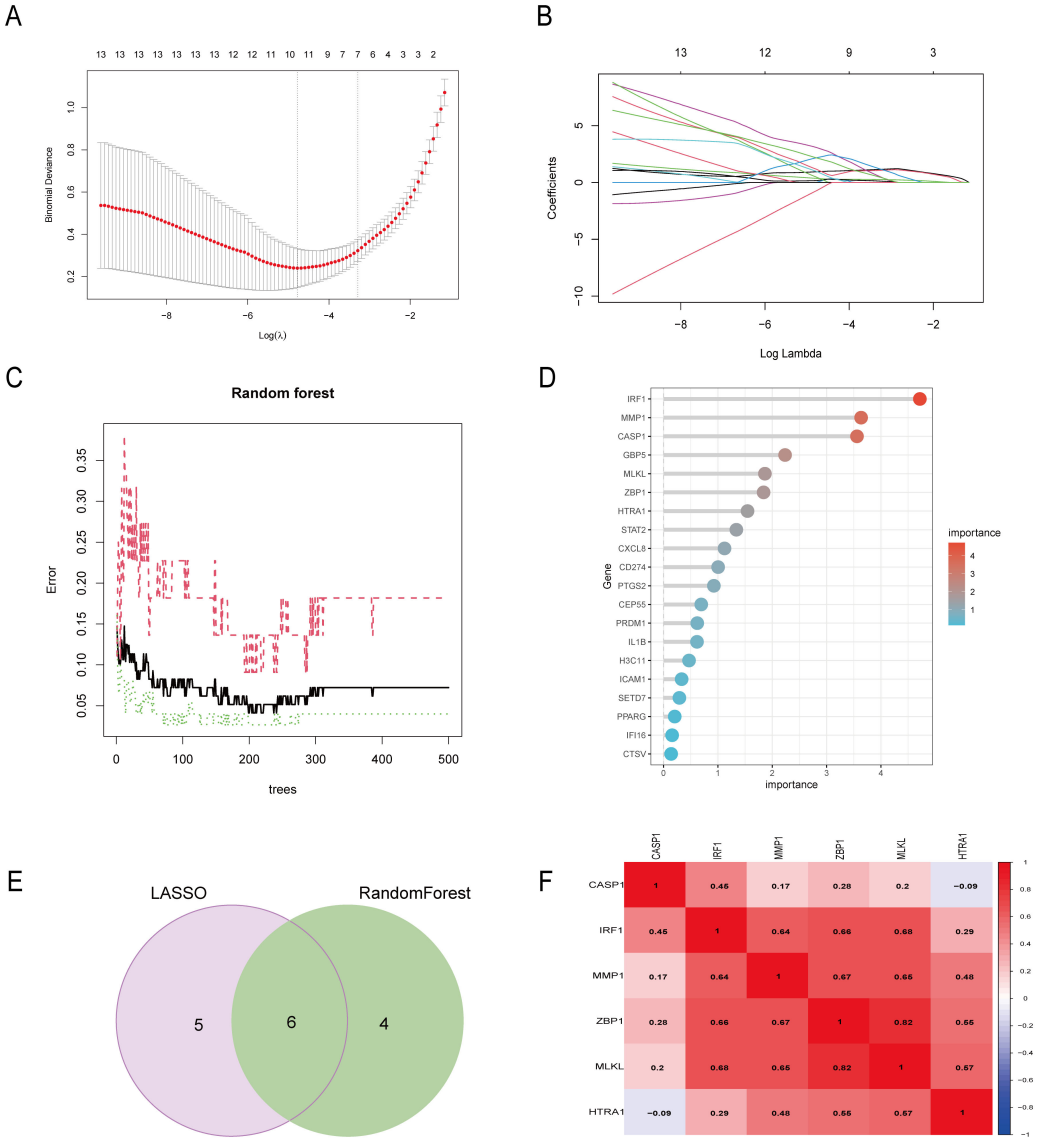
Six DE-PRGs were identified as CD hub genes. **(A, B)** The LASSO algorithm selected 11 CD-related markers. **(C, D)** The RF algorithm selected 10 CD-related markers. **(E)** Six hub genes were obtained from the LASSO and RF algorithms. **(F)** The heatmap shows the correlations of the six hub genes (*CASP1, IRF1, ZBP1, MLKL, MMP1, and HTRA1*) in the CD group from the GSE75214 dataset. The Pearson correlation coefficient spans from –1 (blue) to 1 (red), with –1 representing a strong negative correlation and 1 representing a strong positive correlation between the two genes. DE-PRGs, differentially expressed pyroptosis-related genes; CD, Crohn’s disease; RF, random forest.

**Figure 5:**
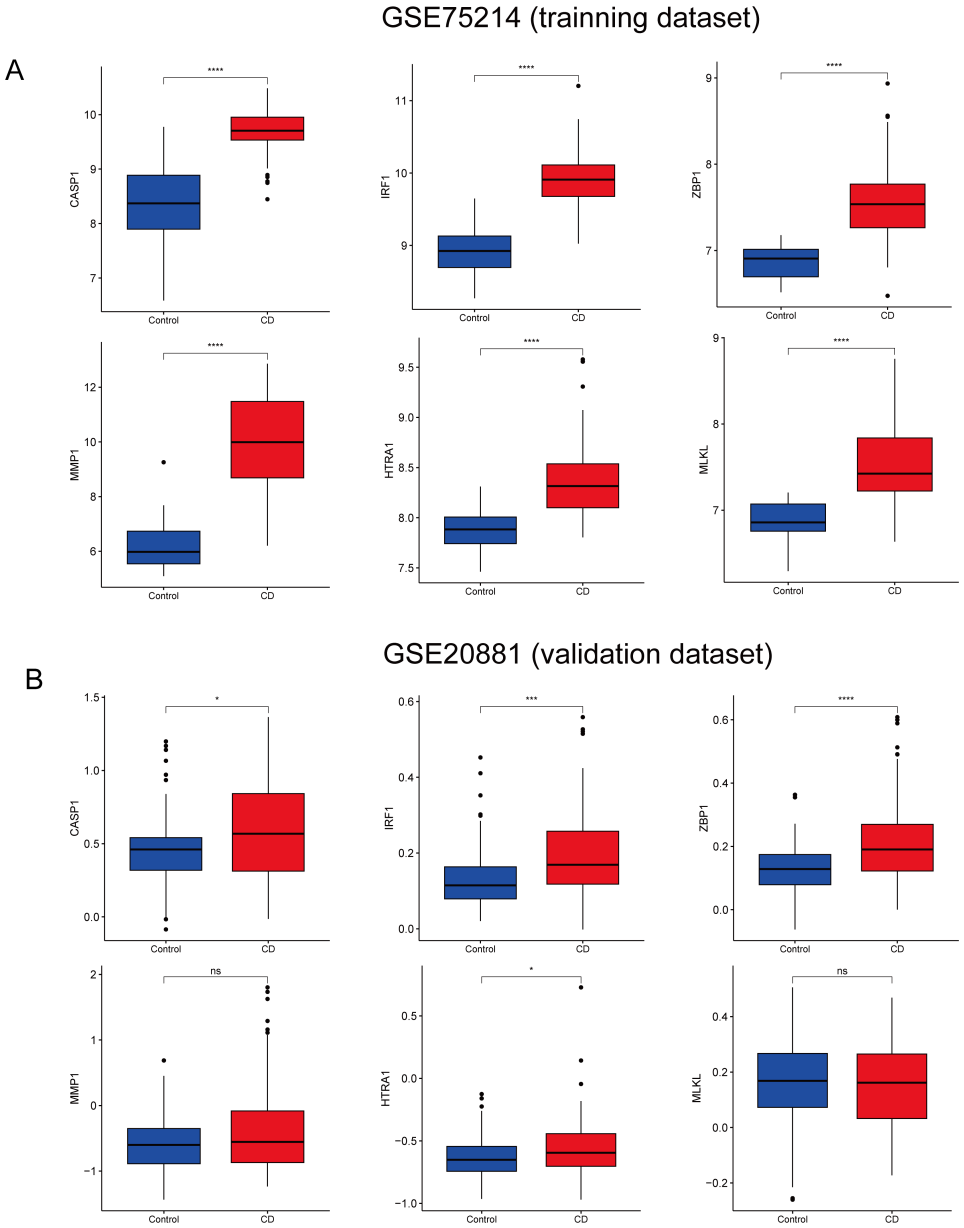
Validation of CD hub genes. **(A)** Boxplot depicting the expression of each hub gene in CD patients and the controls in the GSE75214 dataset. **(B)** Boxplot depicting the expression of each hub gene in CD patients and the controls in the GSE20881 dataset. CD, Crohn’s disease; DE-PRGs, differentially expressed pyroptosis-related genes. ^ns^ *p* > 0.05, *p < 0.05, ***p < 0.001, ****p < 0.0001.

The CD pyroptosis-related hub genes were screened using the LASSO and RF algorithms based on the DE-PRGs (**Supplementary Table S5**). The LASSO algorithm identified 11 DE-PRGs as potential CD biomarkers (**Figures 4A, B**), while the RF algorithm revealed 10 key DE-PRGs as vital biomarkers (**Figures 4C, D**). Overlapping of the algorithm analysis results yielded six genes (*CASP1, IRF1, ZBP1, MLKL, MMP1, and HTRA1*) that were significantly related to CD (**Figure 4E**). Correlation analysis showed predominantly positive relationships among these genes, except for a weak negative correlation between HTRA1 and CASP1 (**Figure 4F**). In the GSE75214 dataset, the CD group exhibited significantly higher expression levels of *CASP1, IRF1, ZBP1, MLKL, MMP1*, and *HTRA1* than the controls (**Figure 5A**). In the GSE20881 dataset, the CD group showed significantly higher expression levels of *CASP1, IRF1, ZBP1*, and *HTRA1* than the controls, while *MLKL* and *MMP1* did not show statistically significant differences (**Figure 5B**). As a result, the expression of four hub genes (*CASP1, IRF1, ZBP1, HTRA1*) was further validated in the single-cell dataset.

### 3.3 Identification of six cell clusters in the single-cell dataset

**Figure 6:**
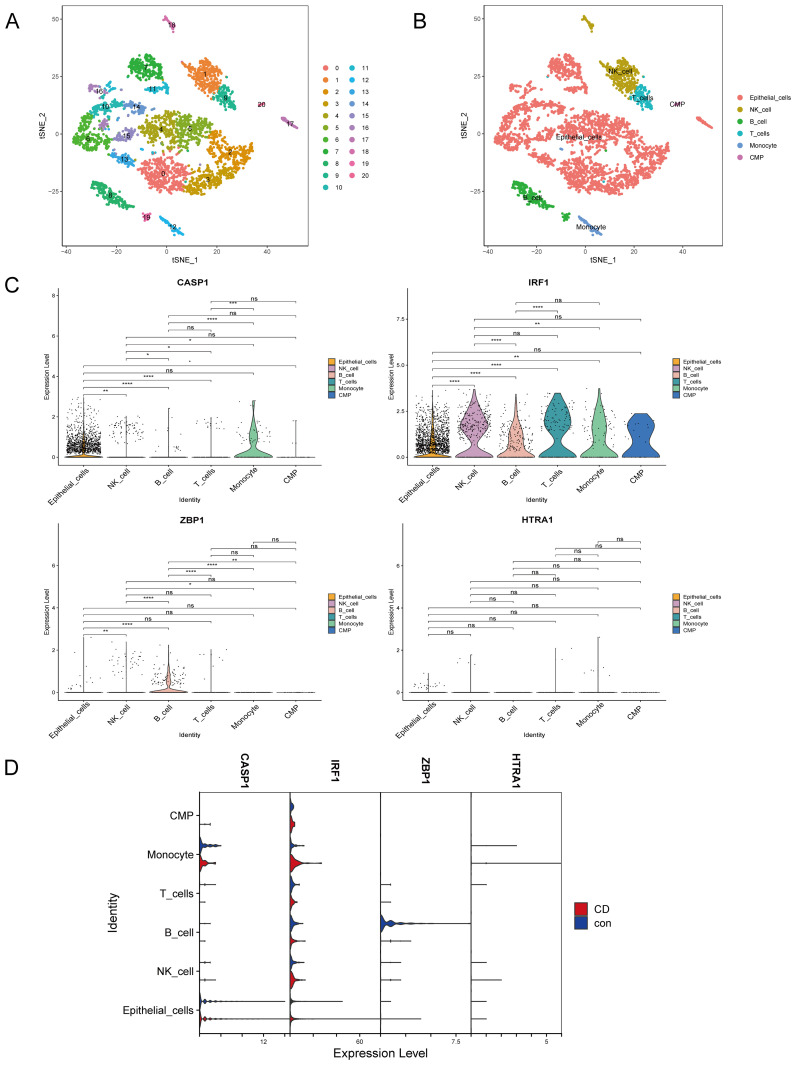
Cell annotation and validation of hub genes in various cell types. **(A)** Reduction of dimensionality by t-SNE. **(B)** Visualization by t-SNE of six cell clusters using marker genes. **(C)** Violin plot depicting CASP1, IRF1, ZBP1, and HTRA1 expression in the six cell clusters. **(D)** Violin plot depicting CASP1, IRF1, ZBP1, and HTRA1 expression in the six cell populations in the CD and control groups. T-SNE, t-distributed stochastic neighbor embedding; CD, Crohn’s disease. ^ns^ *p* > 0.05, *p < 0.05, **p < 0.01, ***p < 0.001, ****p < 0.0001.

The single-cell dataset underwent quality control and normalization for downstream analysis (**Supplementary Figures S2A, B**). The top 2000 HVGs were determined (**Supplementary Figure S2C**). Batch effects between samples were eliminated using the Harmony package (**Supplementary Figure S2D**). Following data integration, PCA dimensionality reduction was performed and revealed that the PC dimension (dim) flattened after 11 (**Supplementary Figure S2E**). Therefore, dim = 11 was selected and the cells were clustered (resolution = 1.2) to obtain 21 subsets (**Figure 6A**, **Supplementary Figure S2F**), where **Supplementary Table S6** lists the marker gene for each cell cluster. Six cell clusters were identified: epithelial cells, natural killer (NK) cells, B cells, T cells, monocytes, and common myeloid progenitors (CMP) (**Figure 6B**, **Supplementary Figure S2G**). As the cells were extracted from intestinal epithelial tissue, most were epithelial cells.

### 3.4 IRF1 and CASP1 were increased in CD IECs in the single-cell dataset

The expressions of the four hub genes (*IRF1, CASP1, ZBP1, HTRA1*) in the different cell types were investigated. *IRF1* was most abundantly expressed in IECs, NK cells, B cells, T cells, monocytes, and common myeloid progenitors (CMP). *CASP1* was mainly expressed in IECs and monocytes, while *ZBP1* and *HTRA1* showed low expression across all cell clusters (**Figure 6C**). Interestingly, *IRF1* and *CASP1* were upregulated in CD IECs compared to the controls (**Figure 6D**).

### 3.5 IECs were strongly linked to monocytes in CD

**Figure 7:**
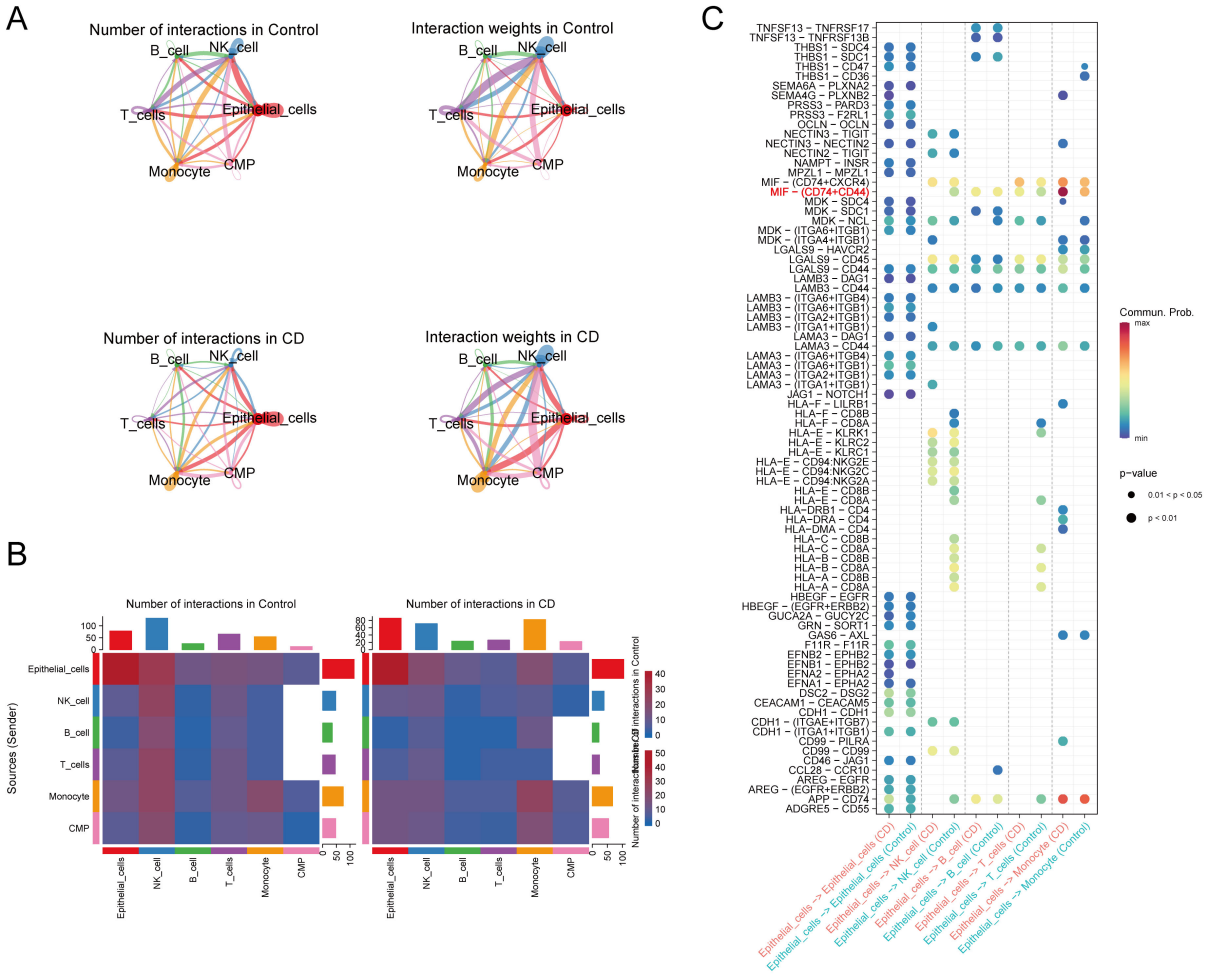
Cell-cell communication. **(A, B)** Analysis of the number and strength of interactions between the different cell clusters in CD and the controls. **(C)** Identification of signaling by comparison of the communication probabilities mediated by ligand-receptor pairs of IECs to other cell types in CD and the controls. CD, Crohn’s disease; IECs, intestinal epithelial cells.

The cellular communication analysis revealed intricate interactions among different cell types (**Figures 7A, B**). In CD, IECs mainly communicated with monocytes and NK cells, with a particularly strong connection to monocytes. Ligand-receptor pair analysis identified an increase in macrophage migration inhibitory factor (MIF) signaling, specifically MIF-(CD74 + CD44) signaling, from IECs to monocytes in CD compared to the controls (**Figure 7C**). In conclusion, our results emphasize the close relationship between IECs and monocytes in CD.

### 3.6 High diagnostic value of IRF1 and CASP1 in CD

**Figure 8:**
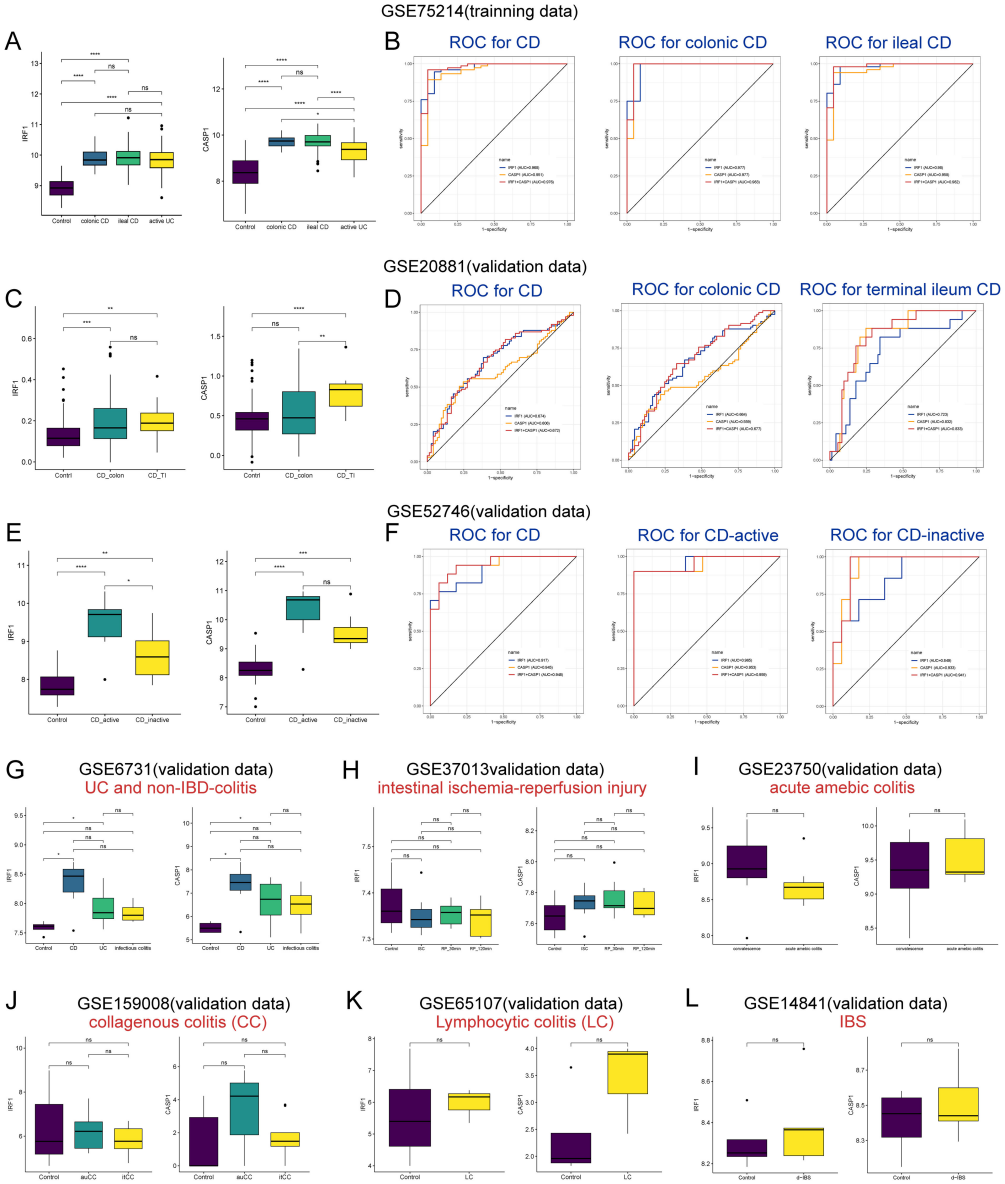
Diagnostic potential of IRF1 and CASP1 in CD subtypes and their expression in other inflammatory disorders. **(A)** The expression of IRF1 and CASP1 in the controls, colonic CD, ileal CD, and active UC groups in the GSE75214 dataset. **(B, D, F)** ROC curve analysis for IRF1 and CASP1 in discriminating and distinguishing CD subtypes in the datasets GSE75214 **(B)**, GSE20881 **(D)**, and GSE52746 **(F)**. **(C)** The expression of IRF1 and CASP1 in the controls, colonic CD, and terminal ileal CD groups in the GSE20881 dataset. **(E)** The expression of IRF1 and CASP1 in the controls, active CD, and inactive CD groups in the GSE52746 dataset. **(G–L)** IRF1 and CASP1 expression in UC **(G)**, infectious colitis **(G)**, intestinal ischemia-reperfusion injury **(H)**, acute amebic colitis **(I)**, CC **(J)**, LC **(K)**, and d-IBS **(L)** were examined across different datasets. CD, Crohn’s disease; UC, ulcerative colitis; TI, terminal ileum; ISC, ischemia; RP, reperfusion; CC, collagenous colitis; auCC, active/untreated collagenous colitis; itCC, inactive/budesonide-treated collagenous colitis; LC, lymphocytic colitis; d-IBS, diarrhea-irritable bowel syndrome. ^ns^ *p* > 0.05, *p < 0.05, **p < 0.01, ***p < 0.001, ****p < 0.0001.

To assess the diagnostic utility of *IRF1* and *CASP1* in CD, as well as to ascertain whether their heightened expression was specific to CD. Their diagnostic efficacy in different CD subtypes and their expressions in other intestinal inflammatory disorders were investigated. In the GSE75214 dataset, both *IRF1* and *CASP1* were significantly upregulated in colonic CD and ileal CD compared to the controls. No significant differences were found in the expression of IRF1 and CASP1 between colonic and ileal CD (**Figure 8A**). *IRF1* and *CASP1* showed robust diagnostic values for CD (AUC 0.968 and 0.951, respectively), colonic CD (AUC 0.977 and 0.977, respectively), and ileal CD (AUC 0.980 and 0.958, respectively) (**Figure 8B**). In the GSE20881 dataset, *IRF1* was notably elevated in colonic CD and terminal ileum CD, and no significant differences were found between colonic and ileal CD. *CASP1* was increased only in terminal ileum CD compared to the controls and colonic CD (**Figure 8C**). *IRF1* and *CASP1* demonstrated superior diagnostic efficacy for CD (AUC 0.674 and 0.606, respectively) and terminal ileum CD (AUC 0.723 and 0.832, respectively), with lower efficacy for colonic CD (AUC 0.664 and 0.559, respectively) (**Figure 8D**). In the GSE52746 dataset, *IRF1* and *CASP1* were significantly upregulated in active and inactive CD compared to the controls (**Figure 8E**). *IRF1* and *CASP1* showed robust diagnostic values for CD (AUC 0.917 and 0.945, respectively), active CD (AUC 0.965 and 0.953, respectively), and inactive CD (AUC 0.849 and 0.933, respectively) (**Figure 8F**). Surprisingly, *IRF1* and *CASP1* were also upregulated in UC compared to the controls (**Figures 8A, G**). Nonetheless, their expression did not significantly increase in infectious colitis, intestinal ischemia-reperfusion injury, amebic colitis, collagenous colitis, lymphocytic colitis, and IBS compared to the controls (**Figures 8H–L**). Overall, our findings highlight the diagnostic significance of *IRF1* and *CASP1* in CD.

### 3.7 IRF1 and CASP1 expression were increased in both CD patients and TNBS-induced colitis rats

**Supplementary Table S7** provides details on participant demographics and baseline characteristics, showing no significant differences in the gender ratio or age distribution between CD patients and the healthy controls. RT-qPCR analysis revealed a significant upregulation of *IRF1* and *CASP1* in CD patients compared to healthy controls (**Figure 1A**). Further analysis found no significant differences in the expression of *IRF1* and *CASP1* between the active and remission phases of CD (**Supplementary Figures S3A, B**). Similarly, there were no significant differences in the expression of *IRF1* and *CASP1* between terminal ileum and colonic subtypes of CD (**Supplementary Figures S3C, D**).

Colitis rats had mucus, watery stool, thin rotten stool, and even bloody stool on day 2 after the TNBS enema. The fecal occult blood test was positive, and there was a progressive decrease in body weight accompanied by a notable increase in the DAI (**Figure 1C**). In the later stage, the self-healing tendency resulted in the gradual decrease of the DAI. Upon sacrifice on day 8, macroscopic evaluation of the colons revealed obvious ulcers and instances of intestinal obstruction with tissue adhesions at the site of obstruction (**Figure 1D**). Histological analysis showed significant thickening of the colonic wall with infiltration of inflammatory cells (**Figure 1E**). The RT-qPCR results demonstrated elevated expression of *Il-1β* and *Tnf-α* mRNA in the colitis rats compared to the controls (**Figure 1F**). Integration of colon DAI scores, macroscopic observations, colon pathology results, and RT-qPCR data confirmed the successful establishment of the TNBS-induced colitis model. Furthermore, both the mRNA and protein expression of Irf1 and Casp1 were significantly upregulated in the colitis rats compared to the controls (**Figure 1G**). These findings from CD patients and colitis rats demonstrate a consistent upregulation of IRF1 and CASP1, indicating a strong association of these genes with CD pathogenesis.

## 4 Discussion

In this study, we have identified IRF1 and CASP1 as potential biomarkers associated with pyroptosis in IECs of CD patients. These markers exhibit upregulation in CD, demonstrating significant diagnostic potential. Epithelial barrier dysfunction plays a key role in mucosal inflammation pathogenesis in IBD (31). IECs are more susceptible to death due to inflammatory drivers, leading to further barrier disruption and flora disorder (11). Pyroptosis is a pro-inflammatory programmed cell death mediated by caspase-1 cleavage of GSDMD and subsequent release of pro-inflammatory mediators (32). Elevated levels of pyroptosis have been observed in ileal and colonic macrophages of CD patients (9). Furthermore, pyroptosis in IECs has been documented (13), with caspase-1 activation playing a pivotal role in this process. The activation of caspase-1 in IECs triggers cell extrusion, further compromising the epithelial barrier and enabling the invasion of luminal microbes and macromolecules into the mucosa, which can initiate inflammatory responses (33). A recent multi-center study identified ileal epithelial cells pyroptosis as a potential predictor of clinical response to vedolizumab in CD patients, highlighting the relevance of pyroptosis in disease management (13).

Our analysis revealed that DE-PRGs were predominantly upregulated in CD. GO and KEGG analyses indicated significant enrichment of DE-PRGs in the inflammasome, NOD-like receptor signaling, and necroptosis pathways. The NOD-like receptor signaling pathway, mediated by NOD-like receptors (NLRs), plays a crucial role in innate immunity and inflammation. NOD2, a member of the NLR family, has been confirmed to be the gene associated with the highest risk for CD (34). Patients with CD who possess NOD2 mutations exhibit a greater susceptibility to ileal involvement and an increased risk of developing fistulae and fibrostenotic phenotypes (35). Loss-of-function mutations in NOD2 contribute to the pathogenesis of CD by disrupting host-microbiota interactions (36) and enhancing pro-inflammatory pathways, including IL-1β, IL-18, and the NLRP3 inflammasome (34). NLRP3 is the most extensively studied inflammasome and is a member of the NLRs family (37). It comprises the NLRP3 protein, apoptosis-associated speck-like protein containing a CARD (ASC), and caspase-1 (38). The single-nucleotide polymorphism rs10733113 within the NLRP3 gene region significantly increases susceptibility to CD (39). Furthermore, animal models have shown that NLRP3 activation promotes pyroptosis and intestinal inflammation in CD (40). Similar to pyroptosis, necroptosis is also a lytic programmed cell death that typically results in an inflammatory response (41). Aberrant activation of necroptosis is also associated with IBD in children and adults (42, 43). Inhibition of necroptosis using specific inhibitors, such as necrostatin-1s, has been demonstrated to mitigate inflammation and tissue damage in experimental models of colitis, suggesting a promising therapeutic strategy for CD (44). Targeting these pathways may offer novel therapeutic approaches for the treatment of CD, offering hope for more effective management of the disease.

In our study, IRF1 and CASP1 demonstrated high diagnostic value for various subtypes of CD, with robust AUC values in distinguishing CD from other conditions. This is in line with the findings of Gong et al. (9) and Tang et al. (45), who observed elevated levels of CASP1 and IRF1 in colonic and ileal CD. However, the diagnostic efficacy of these biomarkers varied between colonic and ileal CD, as well as across different datasets. This variability may be due to genetic heterogeneity and environmental factors that influence the expression patterns of IRF1 and CASP1. Additionally, the sample size and quality of the datasets could also affect the diagnostic accuracy of these biomarkers. Future clinical applications need to consider these variabilities to develop precise diagnostic algorithms, thereby achieving more personalized healthcare. Interestingly, our study detected elevated levels of IRF1 and CASP1 in UC, aligning with the observations of Tang et al. (45) and Li et al. (46), who reported increased expression of these biomarkers in UC. The similar expression patterns in UC could be attributed to the presence of redundant and overlapping pro-inflammatory pathways in both CD and UC (47). However, Christophi et al. (48) reported a significant upregulation of IRF1 in active CD, without a corresponding increase in active UC, indicating a possible divergence in the activation of these pathways between the two conditions. These discrepancies underscore the complexity of IRF1 and CASP1 in IBD and highlight the necessity for further clinical research to dissect their distinct expression profiles in CD and UC.

IRF1, a prominent member of the IRF family, was initially identified as a nuclear factor that activates the type I interferon gene promoter (49). It plays a crucial role in NLRP3 inflammasome formation by inducing ZBP1 transcription during influenza A virus infection (50). IRF1 also regulates the expression of key components necessary for NLRP3 and AIM2 activation after infection, including ZBP1 and guanylate-binding proteins (51). Previous studies have consistently shown elevated IRF1 expression in CD colon tissue compared to normal colon tissue (45), which aligns with our findings. This highlights the significance of IRF1 in CD development.

Encoded by CASP1, caspase-1 is a caspase family member and is activated by inflammasomes to induce pyroptosis (52). Caspase-1 directly cleaves GSDMD and precursor cell factors into pro-IL-1β, which initiates pyroptosis and IL-1β maturation, respectively (32). The classical pyroptosis pathway depends on caspase-1 involvement (53). Increased levels of cleaved caspase-1 protein in macrophages from CD patients (9) and in TNBS-induced colitis mice (54) further support the involvement of caspase-1 in CD pathogenesis.

Pyroptosis was initially described as an innate immune response mediator in macrophages. Macrophage-derived pyroptosis is associated with the inflammatory response in CD (9). We hypothesized that the crosstalk between macrophages and IECs regarding pyroptosis could influence CD pathogenesis. Macrophages are derived from monocytes (55). Our findings suggest a potential crosstalk between IECs and monocyte/macrophage pyroptosis through the MIF-(CD74 + CD44) pathway activation, influencing CD pathogenesis. MIF is a key cytokine in many inflammatory diseases, including IBD (56). MIF signaling is initiated by binding to CD74/CD44, mediating most proliferative and inflammatory effects (57, 58). MIF promotes kidney tubular cell pyroptosis by controlling the NF-κB–NLRP3 pathway in sepsis-induced acute kidney injury (59). This suggests that MIF could play a broader role in inflammatory processes, including those in CD. The interaction between IECs and monocytes, particularly in the context of pyroptosis, may play a significant role in intestinal inflammation in CD. However, further experimental validation is needed to elucidate the specific mechanisms involved.

While our study provides valuable insights into the roles of IRF1 and CASP1 in CD pathogenesis, some limitations need to be addressed. Firstly, the validation samples in our study were obtained from a single center with a small sample size. Large-scale and multicenter studies are still needed to confirm the results in the future. Furthermore, the elevated levels of IRF1 and CASP1 observed in UC suggest that these biomarkers may not be entirely specific to CD. It will be crucial to validate the expression patterns of IRF1 and CASP1 across a wider range of clinical samples and to identify additional biomarkers that can more accurately differentiate CD from UC. Expanding the scope of research to multiple centers will enhance the identification and validation of such biomarkers, ultimately contributing to more precise clinical diagnostics and decision-making.

In conclusion, our study has pinpointed IRF1 and CASP1 as promising pyroptosis-related markers in IECs of CD. These genes displayed significant upregulation and strong diagnostic value across various CD subtypes. Moreover, our findings suggest potential crosstalk between IECs and monocyte/macrophage pyroptosis through the MIF-(CD74 + CD44) pathway activation, shedding light on the intricate mechanisms influencing CD pathogenesis. The potential role of IRF1 and CASP1 in CD highlights their significance for future research and clinical applications in the management of this multifaceted disease.

## Data Availability

The original contributions presented in the study are included in the article/**Supplementary Material**. Further inquiries can be directed to the corresponding author.
