# Supplementary figures and images for: Elevated levels of IRF1 and CASP1 as pyroptosis-related biomarkers for intestinal epithelial cells in Crohn’s disease
Source: Front Immunol. 2025 Feb 13;16:1551547. doi: 10.3389/fimmu.2025.1551547 (PMC11865233; doi:10.3389/fimmu.2025.1551547)

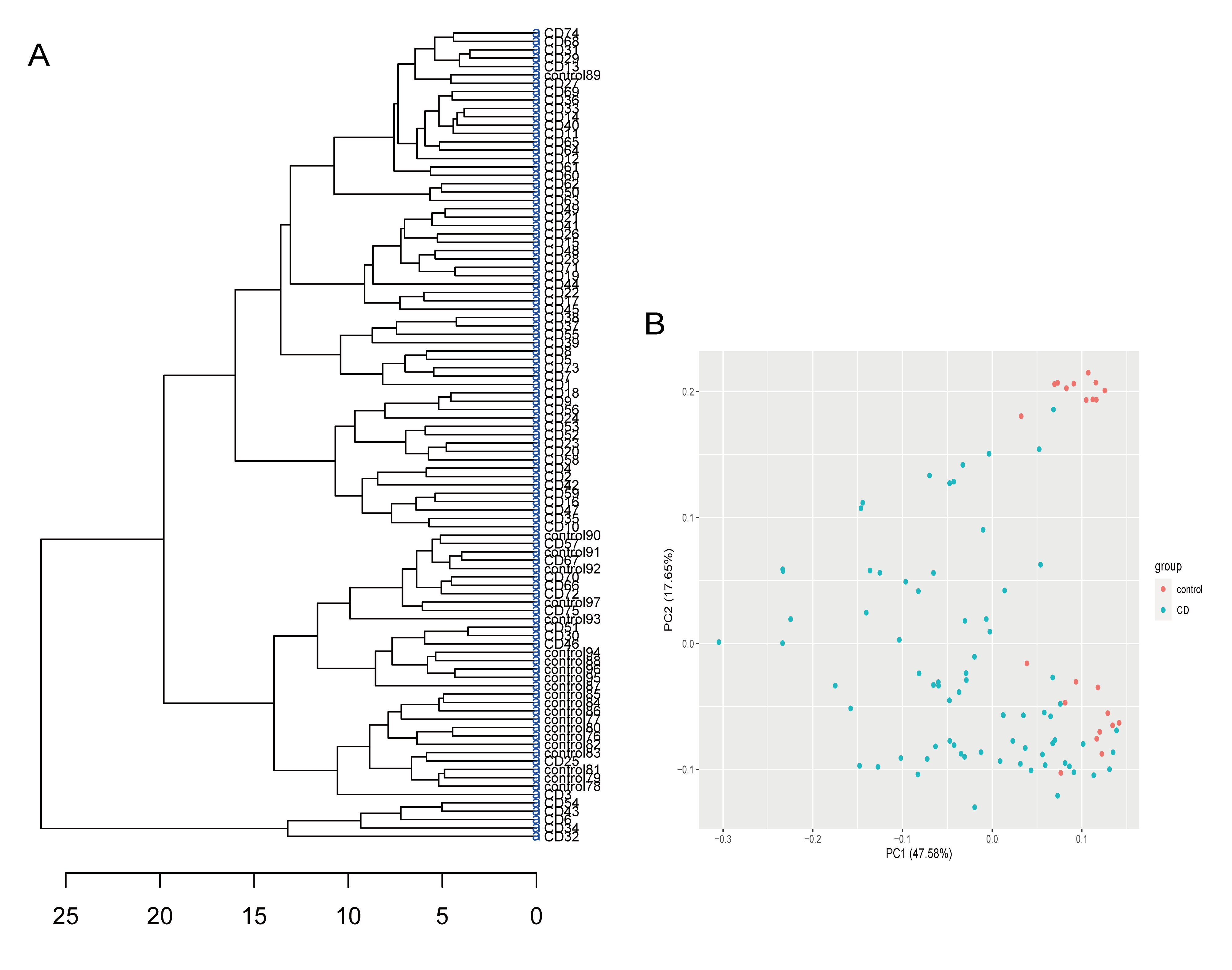

Supplement: Supplementary file 1 [file Image1.tif]

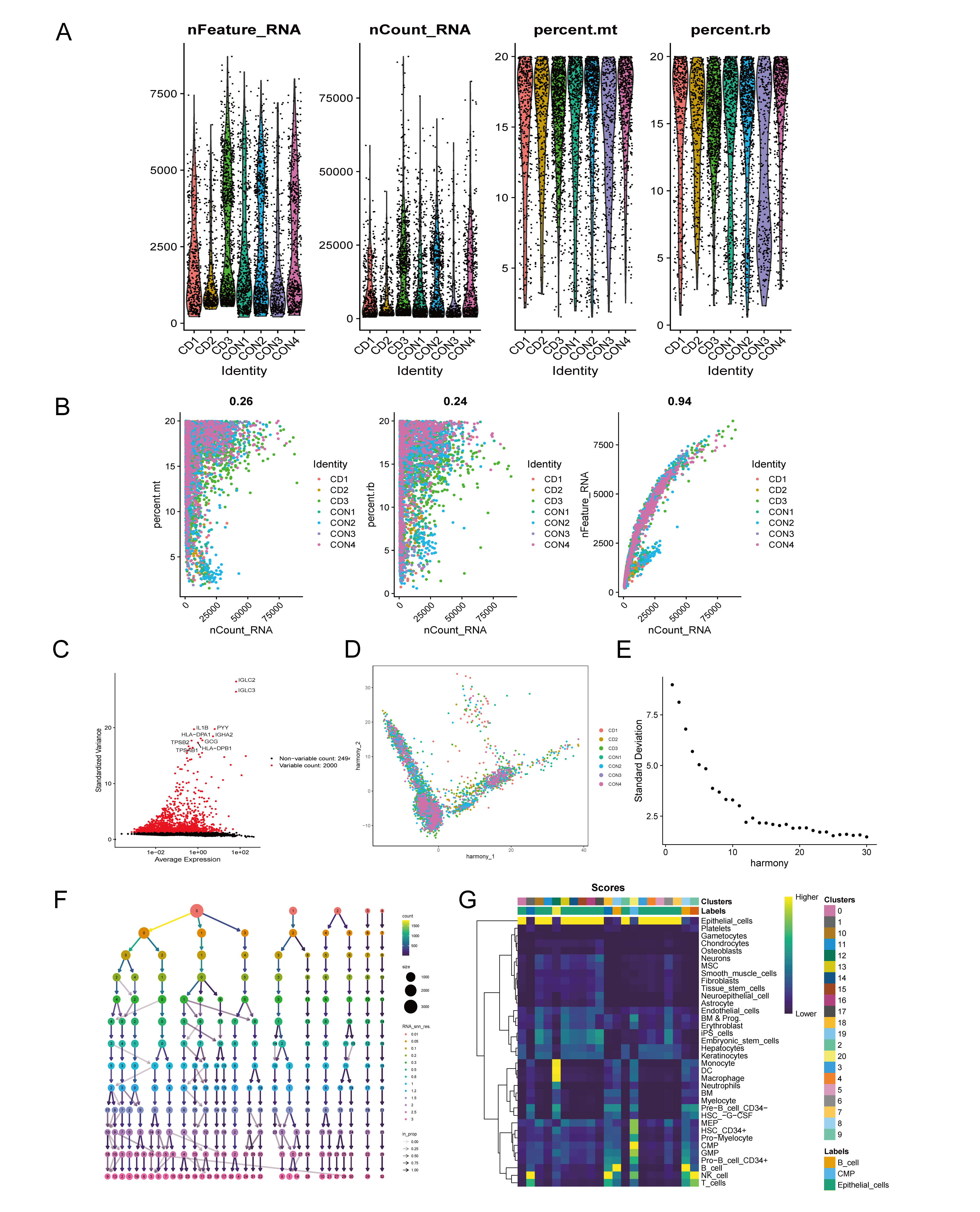

Supplement: Supplementary file 2 [file Image2.tif]

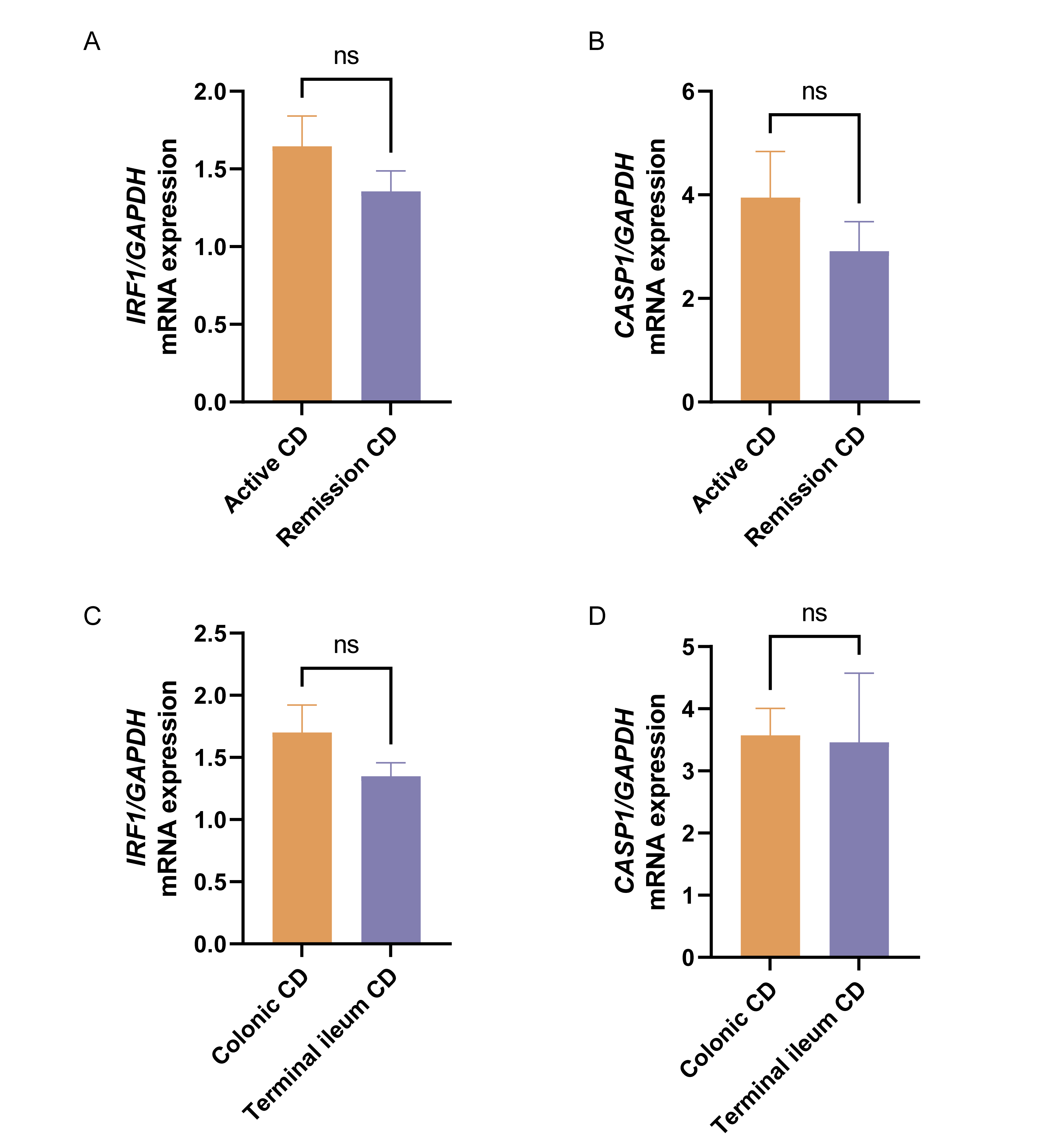

Supplement: Supplementary file 3 [file Image3.tif]
