# Supplementary material for: Elevated levels of IRF1 and CASP1 as pyroptosis-related biomarkers for intestinal epithelial cells in Crohn’s disease
Source: Front Immunol. 2025 Feb 13;16:1551547. doi: 10.3389/fimmu.2025.1551547 (PMC11865233; doi:10.3389/fimmu.2025.1551547)
Supplement: Supplementary file 4 [file Table1.docx]

Table S1 Summary of GEO datasets and sample information.

| GEO accession | Sample source | Samples | | | | |
| --- | --- | --- | --- | --- | --- | --- |
| GSE75214 | Intestinal mucosal biopsy | Control  (n = 22) | Active colonic CD (n = 8) | Active ileal CD (n = 67) | | UC  (n = 74) |
| GSE20881 |  | Control  (n = 73) | Colonic CD  (n = 82) | Terminal ileum CD  (n = 17) | |  |
| GSE52746 |  | Control  (n = 17) | Active CD  (n = 10) | Inactive CD  (n = 7) | |  |
| GSE6731 |  | Control  (n = 4) | CD  (n = 7) | UC  （n = 9） | | infectious colitis (n = 4) |
| GSE37013 |  | Control  (n = 7) | Ischaemia  (n = 7) | Reperfusion 30min (n = 7) | | Reperfusion 120min  (n = 7) |
| GSE23750 |  | Convalescence  (n = 8) | Acute amebic colitis (n = 8) |  | |  |
| GSE159008 |  | Control  （n = 13） | auCC  (n = 9) | itCC  (n = 9) | |  |
| GSE65107 |  | Control  （n = 4） | LC  (n = 4) |  |  | |
| GSE1484 |  | Control  （n = 4） | d-IBS  (n = 5) |  |  | |

CD, Crohn’s disease; UC, ulcerative colitis; ISC, Ischemia; RP, reperfusion; auCC, active/untreated collagenous colitis; itCC, inactive/budesonide-treated collagenous colitis; LC, lymphocytic colitis; d-IBS, diarrhea-irritable bowel syndrome.
