# Supplementary material for: Elevated levels of IRF1 and CASP1 as pyroptosis-related biomarkers for intestinal epithelial cells in Crohn’s disease
Source: Front Immunol. 2025 Feb 13;16:1551547. doi: 10.3389/fimmu.2025.1551547 (PMC11865233; doi:10.3389/fimmu.2025.1551547)
Supplement: Supplementary file 10 [file Table7.docx]

**Supplementary Table S7** Summary of colonic biopsies samples information

|  | Control | CD | *p* |
| --- | --- | --- | --- |
| n | 12 | 12 |  |
| Age（mean ± SEM） | 45.08±4.40 | 37.58±3.14 | 0.17 |
| Sex | Male (n = 5)  Female (n = 7) | Male (n = 7)  Female (n = 5) | >0.414 |
| Disease activity | - | Active (n = 7)  Remission (n = 5) | - |
| Disease severity | - | Mild (n = 2)  Moderate (n = 4)  Severe (n = 1) |  |
| Sample locations | Ileocecal Region (n = 1)  Terminal Ileum (n = 2)  Ascending Colon (n = 3)  Transverse Colon (n = 2)  Descending Colon (n = 1)  Sigmoid Colon (n = 2)  Rectum (n = 1) | Ileocecal Region (n = 4)  Terminal Ileum (n = 2)  Ascending Colon (n = 2)  Transverse Colon (n = 1)  Descending Colon (n = 0)  Sigmoid Colon (n = 2)  Rectum (n = 1) |  |

CD, Crohn’s disease; SEM, standard error of the mean.
