# Supplementary material for: Elevated levels of IRF1 and CASP1 as pyroptosis-related biomarkers for intestinal epithelial cells in Crohn’s disease
Source: Front Immunol. 2025 Feb 13;16:1551547. doi: 10.3389/fimmu.2025.1551547 (PMC11865233; doi:10.3389/fimmu.2025.1551547)
Supplement: Supplementary file 11 [file Table8.docx]

Supplementary Material

# Supplementary Figures and Tables

## Supplementary Figures

**Supplementary Figure 1.** Evaluation of the dissimilarity between each sample. **(A)** Cluster plot. **(B)** PCA score plot.

**Supplementary Figure 2.** Single-cell processing and cell clustering. **(A)** Gene features, counts, and percentages of mitochondrial genes and red blood cell genes of the sample. **(B)** Correlation between sample gene counts with mitochondrial gene, red blood cell gene, and feature genes. **(C)** Gene scatter plot shows 2000 HVGs and the top 10 HVGs. **(D)** Harmony package removal of the batch effect between different samples. **(E)** Elbow Plot of single-cell dataset. **(F)** Phylogenic tree of the 21 clustered subpopulations (resolution = 1.2). **(G)** Identification of six cell clusters using different annotation methods.

**Supplementary Figure 3.** Expression levels of IRF1 and CASP1 in active and remission CD, as well as in colonic and terminal ileal CD. (**A-B**) No significant differences were observed in the expression of IRF1 and CASP1 between active and remission stages of CD. **(C-D)** No significant differences were observed in the expression of IRF1 and CASP1 between colonic and terminal ileal subtypes of CD. CD, Crohn’s disease.

## Supplementary Tables

**Supplementary Table S1** Summary of GEO datasets and sample information.

**Supplementary Table S2** A total of 253 protein-coding PRGs.

**Supplementary Table S3** The primer sequences f or RT-qPCR used in this study.

**Supplementary Table S4** Differentially expressed of PRGs in the GSE75214 dataset.

**Supplementary Table S5** The LASSO and RF algorithm identified key DE-PRGs as CD biomarkers.

**Supplementary Table S6** The marker gene for each cell cluster.

**Supplementary Table S7** Summary of colonic biopsies samples information.
